# Tumor-like periungual cutaneous sporotrichosis in an endemic area^؆^

**DOI:** 10.1016/j.abd.2026.501397

**Published:** 2026-07-01

**Authors:** Mathias Ferreira, Thiago Vilas Boas, Kananda Kesye Sousa Nunes, Guilherme Caldas de Souza, Carolina Talhari

**Affiliations:** a Postgraduate Program in Applied Sciences to Dermatology, Universidade do Estado do Amazonas, Manaus, AM, Brazil; b Faculty of Medicine, Universidade Federal do Amazonas, Manaus, AM, Brazil

Dear Editor,

Sporotrichosis is the most prevalent subcutaneous mycosis in tropical and subtropical areas, caused by fungi of the genus *Sporothrix*,^1^ whose transmission occurs predominantly through bites or scratches from infected animals in urban areas of endemic regions,^2^ such as the Amazon region.^3^

The clinical forms of cutaneous sporotrichosis are divided into: lymphocutaneous, fixed cutaneous, and disseminated. The lymphocutaneous form is the most common, characterized by ulcers, classically accompanied by lymphangitis. It usually affects the upper limbs of adult patients and the face in children.^1^, ^4^ In forms without lymphangitis, such as the fixed cutaneous form, the diagnosis may be less remembered.^5^

This report describes a patient with localized cutaneous sporotrichosis with an exuberant and atypical tumor presentation, diagnosed in an endemic area.

The patient is a 44-year-old female, a florist for 15 years, residing in Manaus, Amazonas, who was previously healthy. For two weeks, she presented with a painful, progressively growing lesion on the second finger of her left hand, following trauma from a plant thorn. The patient denied contact with sick animals. On physical examination, an ulcerated, purplish, friable tumor was observed, covered by fibrinous material and involving the entire distal extremity, associated with microvesicles at the base of the lesion, without lymphangitis (Fig. 1). She had received cephalexin 500 mg every 6 hours for seven days, without clinical improvement.

**Fig. 1:**
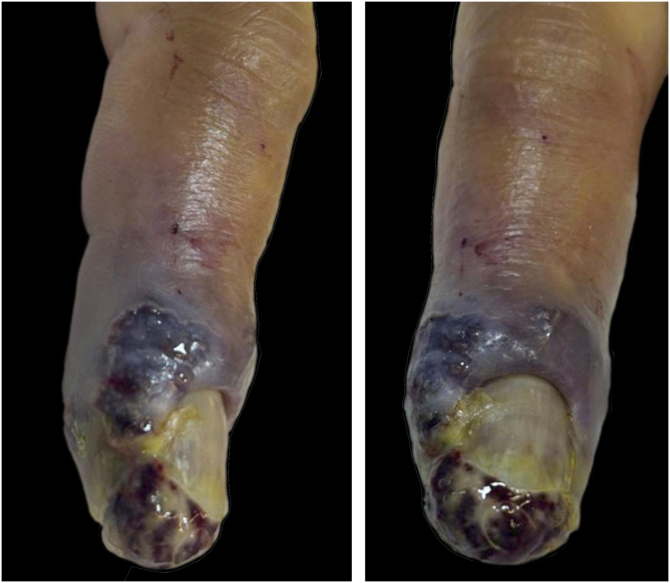
A purplish, ulcerated, friable, and vegetating nodule covered by fibrinous exudate in the periungual region.

An incisional biopsy was performed, and fragments were sent for culture and histopathology. The histological examination revealed a suppurative granulomatous process (Fig. 2), with no fungi detected by PAS. The bacterial culture was negative, while the fungal culture isolated *Sporothrix spp* (Fig. 3). Real-time polymerase chain reaction from culture material revealed *Sporothrix brasiliensis*. Itraconazole, 200 mg/day, was started, with significant improvement in pain and significant lesion regression in three weeks of use. After four months of treatment, there was complete resolution of the tumor (Fig. 4).

**Fig. 2:**
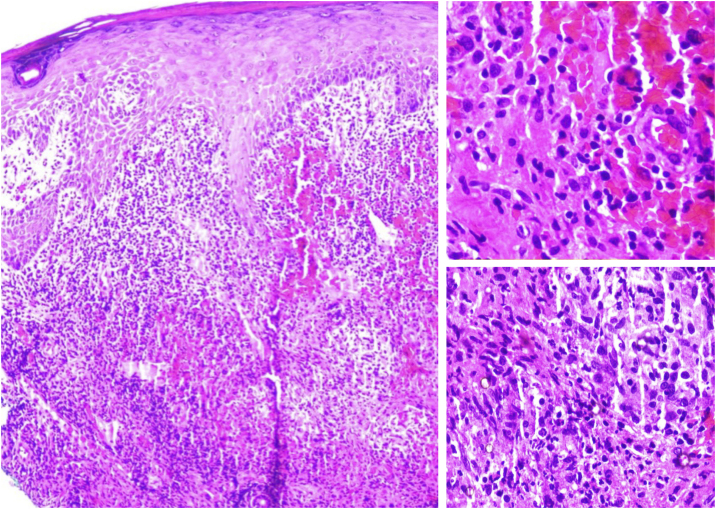
Epidermal hyperplasia and granulomatous infiltrate with neutrophils (left. Hematoxylin & eosin, ×4). Infiltrate of epithelioid cells (upper right. Hematoxylin & eosin, ×40). Neutrophil microabscess in the dermis (lower right. Hematoxylin & eosin, ×40).

**Fig. 3:**
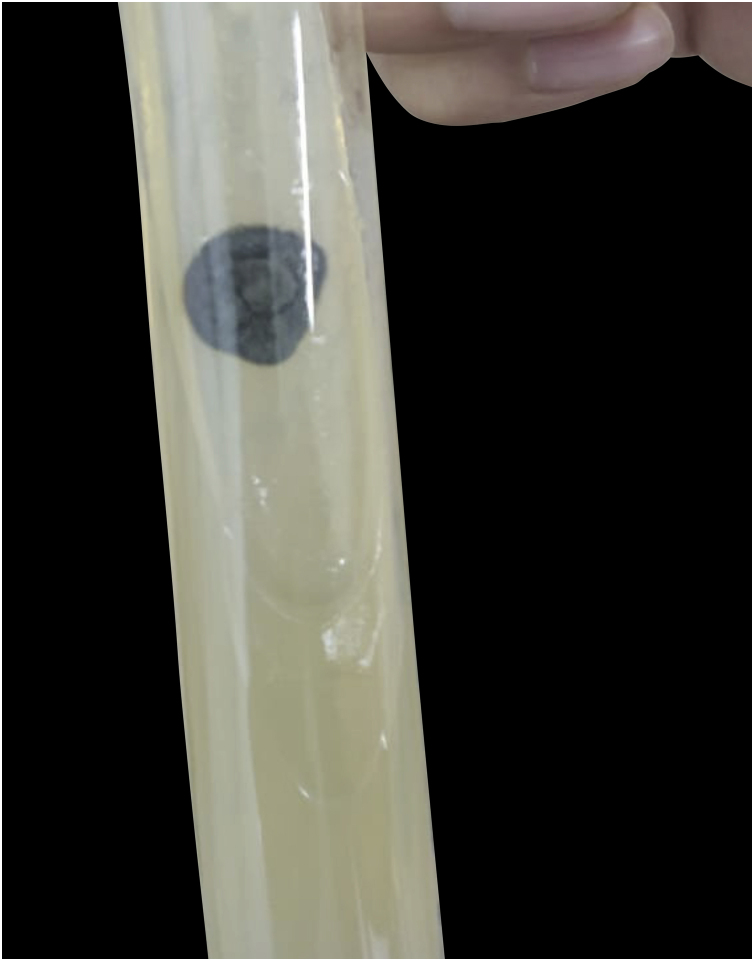
Culture for *Sporothrix sp*. at 25°, after 6 weeks.

**Fig. 4:**
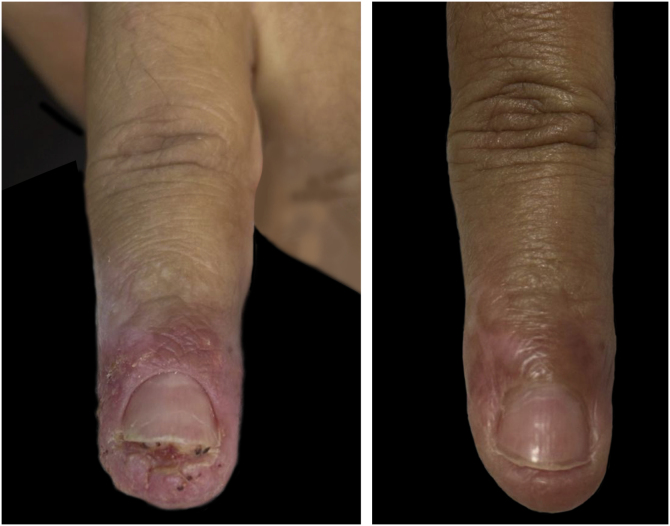
Patient clinical improvement after three weeks of itraconazole (left). Complete lesion resolution after four months (right).

The presence of a painful tumor-like lesion in the periungual region suggests neoplastic diseases such as glomus tumor, squamous cell carcinoma, and melanoma, as well as other diseases such as verruca vulgaris, chronic paronychia, and subungual exostosis.^5^, ^6^ The classic presentation of cutaneous sporotrichosis consists of a nodular lesion at the inoculation site, which may evolve into ulceration and lymphocutaneous dissemination, most frequently affecting the upper limbs and occasionally involving the fingers and periungual regions. Chronic, slow-healing ulcers on the thumb and periungual region have been described, especially after minimal trauma, with subsequent development of progressive erythema, edema, and persistent pain.^7^ Rarely, sporotrichosis can induce pseudoepitheliomatous hyperplasia, resulting in a hyperplastic or tumor-like lesion.^8^

Between 2021 and 2022, there was a 304% increase in the number of cases of zoonotic sporotrichosis in Manaus. Between 2022 and 2023, this increase was 349%. The main animal involved in transmission is the cat. As in other endemic areas, the main etiological agent is *Sporothrix brasiliensis.*^3^, ^9^

In the present case, the occurrence of cutaneous infection by *S. brasiliensis* without a history of contact with sick animals is epidemiologically plausible and clinically relevant. The patient is a florist, with daily handling of flowers, branches, thorns, mosses and soil, a circumstance that implies repeated microtraumas by organic material and, therefore, potential non-zoonotic inoculation. In endemic scenarios, the high fungal load resulting from the epidemic in felines favors the environmental dissemination of the agent (soil, plant matter and objects), which increases the probability of traumatic implantation from contaminated material and explains autochthonous cases in individuals without direct exposure to cats.^10^

In endemic regions, especially in the Amazon region, the possibility of sporotrichosis should always be considered in the differential diagnosis of exuberant, painful, or tumor-like skin lesions in the periungual region, even in the absence of contact with sick animals, especially when there is a history of trauma with organic material. The atypical clinical presentation, such as the tumor-like form, can mimic neoplasms or other infectious and inflammatory dermatoses, requiring a high degree of clinical suspicion and mycological confirmation. Early recognition and appropriate treatment are fundamental to avoiding local complications and ensuring complete resolution of the condition. This report reinforces the importance of clinical surveillance in areas of active transmission, as well as the integration between dermatology and mycology services in the identification and management of unusual cases of cutaneous sporotrichosis.

## Authors' contributions

Mathias Ferreira: Design and planning of the study; drafting and editing of the manuscript; critical review of the manuscript; critical review of the literature; approval of the final version of the manuscript.

Thiago Vilas Boas: Drafting and editing of the manuscript; critical review of the manuscript.

Kananda Kesye Sousa Nunes: Critical review of the manuscript; critical review of the literature.

Guilherme Caldas de Souza: Intellectual participation in the propaedeutic and/or therapeutic conduct of the studied cases.

Carolina Talhari: Design and planning of the study; drafting and editing of the manuscript; critical review of the manuscript; approval of the final version of the manuscript.

## Financial support

None declared.

## Research data availability

Does not apply.

## Conflicts of interest

None declared.
